# Supplementary material for: WordCommentsAnalyzer: A windows software tool for qualitative research
Source: F1000Res. 2018 Sep 4;7:536. Originally published 2018 May 3. [Version 2] doi: 10.12688/f1000research.14819.2 (PMC6137411; doi:10.12688/f1000research.14819.2)
Supplement: Supplementary file 3 [file f1000research-7-17614-s0002.tgz › b2a79496-4ada-47c4-9682-a99ce867836d.zip!/Eight Iranian Health Journals/Journal of Health System Research _2.docx]

© Journal of Health System Research . This work is licensed under CC BY-NC 4.0 (https://creativecommons.org/licenses/by-nc/4.0/).

Volume & Issue: دوره 13، شماره 4، 1396

Title: ارزیابی شاخص‌های گرمایی محیطی، فیزیولوژیک و ادراکی در کارگران یک معدن زیرزمینی

http://hsr.mui.ac.ir/index.php/jhsr/article/view/2778

مقدمه: گرما به عنوان عامل زیان‌آوری در محیط‌های کاری شناخته شده است که باعث ایجاد تغییرات فیزیولوژیک در افراد می‌شود. هدف از انجام مطالعه حاضر، ارزیابی شاخص‌های گرمایی محیطی، فیزیولوژیک و ادراکی در یک معدن زیرزمینی بود. روش‌ها: این پژوهش تحلیلی- مقطعی، بر روی 64 نفر از کارگران مرد یک معدن زیرزمینی سرب و روی در فصل تابستان انجام گرفت. شاخص‌های استرین فیزیولوژیک (Physiological strain index یا PSI) و شاخص دمای تر گوی‌سان (Wet bulb globe temperature یا WBGT) به طور هم‌زمان اندازه‌گیری شد و همچنین، شاخص نمره استرین حرارتی (Heat strain score index یا HSSI) با استفاده از پرسش‌نامه مربوط تکمیل گردید. داده‌های به دست آمده در نرم‌افزار SPSS مورد تجزیه و تحلیل قرار گرفت. یافته‌ها: میانگین شاخص‌های PSI، WBGT و HSSI به ترتیب برابر با 11/2، 08/22 و 55/13 بود و نشان دهنده آن است که محیط معدن دارای استرس حرارتی کمی می‌باشد. میانگین شاخص PSI در سطوح خطر شاخص HSSI دارای اختلاف معنی‌داری بود. بر اساس نتایج آزمون همبستگی Pearson، بین شاخص‌های WBGT و PSI ارتباط معنی‌داری مشاهده نشد (791/0 = P). همچنین، همبستگی خوبی بین شاخص‌های HSSI و PSI وجود داشت (001/0 > P، 859/0 = r). نتیجه‌گیری: با توجه به محدودیت‌های استفاده از شاخص PSI، می‌توان از شاخص HSSI به عنوان يك ابزار غربالگري در ارزیابی سطح استرین گرمایی در کارگران معادن زیرزمینی استفاده نمود.

Title: بررسی میزان پراکسید هیدروژن در روغن‌های مصرفی در مغازه‌های اغذیه فروشی شهر کرمانشاه در سال 1395

http://hsr.mui.ac.ir/index.php/jhsr/article/view/3162

مقدمه: رشد روزافزون مصرف غذاهای فوری مانند انواع ساندویچ، همبرگر و... در بین نوجوانان و جوانان و روش طبخ این غذاها که اغلب به شکل سرخ کرده می‌باشد، باعث آسیب به سلامتی می‌شود؛ چرا که سرخ كردن زياد روغن خوراکی منجر به هيدروليز، اكسيداسيون و پليمريزاسيون روغن مي‌گردد. تولید راديكال‌هاي آزاد ناشی از سرخ كردن زياد روغن از جمله علل سرطان، بيماري‌هاي التهابي و گوارشی، آترواسكلروز، پيري و نظاير آن مي‌باشد. هدف از انجام مطالعه حاضر، بررسی میزان پراکسید در روغن‌های مصرفی اغذیه فروشی‌های شهر کرمانشاه در سال 1395 بود. روش‌ها: این پژوهش به روش توصیفی- مقطعی در اغذیه فروشی‌های شهر کرمانشاه انجام شد. بدین ترتیب، بر اساس استاندارد 493 مؤسسه استاندارد وتحقيقات صنعتي ايران، 81 نمونه از روغن‌های مصرفی برداشت شد و پس از انتقال به آزمايشگاه، عدد پراكسيد آن‌ها طبق استاندارد شماره 4179 اندازه‌گیری گردید و مقادیر به دست آمده مورد بررسی قرار گرفت. یافته‌ها: از مجموع 81 نمونه مورد بررسی، 59 درصد (48 نمونه) از روغن‌ها قابل مصرف و در حد استاندارد و 41 درصد (33 نمونه) آن‌ها غیر قابل مصرف و بالاتر از حد استاندارد مؤسسه استاندارد و تحقيقات صنعتي ايران (5 میلی اکی‌والان در کیلوگرم) بود. علاوه بر این، کمترین و بیشترین عدد اکسایش پراکسید به ترتیب صفر و 29 میلی اکی‌والان در کیلوگرم به دست آمد. نتیجه‌گیری: با توجه به این که حدود نیمی از روغن‌های خوراکی مورد بررسی در مطالعه حاضر، مقدار پراکسید بیشتر از حد مجاز داشت؛ بنابراین، لزوم نظارت بیشتر، اطلاع‌رسانی به مصرف کنندگان و اجرای دوره‌های آموزشی و بهداشتی برای شاغلان این بخش امری ضروری به نظر می‌رسد.

Title: بررسی میزان تأثیر دوره‌های آموزشی ضمن ‌خدمت بر کاهش استرس شغلی کارکنان دانشگاه علوم پزشکی اصفهان

http://hsr.mui.ac.ir/index.php/jhsr/article/view/3229

مقدمه: دوره‌هاي آموزشي ضمن ‌خدمت، كاركنان سازمان را در حل مشكلات و اخذ تصميمات شغلی توانمند می‌سازد. هنگامی که نيازهاي شغلي با توانايي‌های فرد همخواني نداشته باشد، استرس شغلي رخ مي‌دهد که منجر به بروز تأثیرات منفی بر سلامت جسمي و رواني، کاهش کارايي و عدم بهره‌وري در سازمان می‌گردد. مطالعه حاضر با هدف بررسی تأثیر دوره‌های آموزش ضمن‌ خدمت بر کاهش استرس شغلی کارکنان صورت گرفت. روش‌ها: این پژوهش به صورت توصيفي- تحليلي بر روي 120 نفر از كاركنان دانشگاه علوم پزشكي اصفهان با نمونه‌گيري تصادفي انجام شد. ابزار جمع‌آوري داده‌ها، پرسش‌نامه‌های ضمن ‌خدمت ستوده و استرس شغلی  Davis به ترتیب با ضریب Cronbach¢s alpha برابر با 70/0 و 90/0 بود. داده‌ها در نرم‌افزار SPSS مورد تجزیه و تحلیل قرار گرفت. یافته‌ها: 5/35 درصد شركت‌ کنندگان مرد و 5/64 درصد زن بودند. میانگین نمره استرس شغلی کارکنان، 3/11 ± 4/16 به دست آمد. بر اساس آزمون همبستگی Pearson، بین نمره استرس شغلی با نوع استخدام، دلایل شرکت در دوره‌ها و مزایای آن‌ها ارتباط معنی‌داری مشاهده شد (050/0 > P). بین نمره استرس شغلی و تأثیرات دوره‌ها (رضایتمندی) رابطه معکوس (208/0- = r، 020/0 = P) و با میزان غیبت در طول سال رابطه مستقیمی وجود داشت (424/0 = r، 001/0 > P). نتیجه‌گیری: کسب مهارت‌هاي فني و عملياتي، از مهم‌ترین نیازهای کارکنان محسوب مي‌شود. بنابراین، ضروري است سازمان در جهت ارتقای شغلی کارکنان با به كارگيري راهكارهاي حمايتي، از تأثيرات درازمدت استرس شغلي بر عملکرد و انگیزش آنان پيشگيري نماید.

Title: بررسی عملکرد روش رقیق‌سازی برای کاهش نیترات آب در شهرستان فریدن

http://hsr.mui.ac.ir/index.php/jhsr/article/view/3068

مقدمه: شهرستان فریدن با جمعیتی حدود 84 هزار نفر و با آب و هوای سرد و کوهستانی، در شمال غربی استان اصفهان واقع شده است و به دلیل وسعت زیاد زمین‌های کشاورزی و استفاده بی‌رویه از کودهای شیمیایی (به خصوص کودهای ازته) و خشکسالی، میزان نیترات آب آشامیدنی در برخی از مناطق این شهرستان بالاتر از حد مجاز می‌باشد. نیترات را می‌توان به روش‌های شیمیایی، بیولوژیکی و فیزیکی از آب حذف کرد. از این‌رو، پژوهش حاضر با هدف کاهش نیترات و رساندن آن به مقدار مجاز صورت گرفت و بدین منظور از روش رقیق‌سازی استفاده گردید. روش‌ها: این مطالعه از نوع تجربی مداخله‌ای بود که در آن عملکرد روش رقیق‌سازی جهت کاهش نیترات آب در شهرستان فریدن مورد بررسی قرار گرفت. از این روش می‌توان به عنوان روش قابل اعتماد و اقتصادی جهت کاهش نیترات آب خوراکی استفاده کرد. یافته‌ها: پس از رقیق‌سازی نمونه‌های جمع‌آوری شده از چاه‌های مختلف شهرستان فریدن، آزمایش رقیق‌سازی انجام شد و مشخص گردید که نیترات آب به دست آمده برابر با میانگین نیترات دو آب اولیه بود و مقدار آن به کمتر از حد مجاز رسید. نتیجه‌گیری: حذف نیترات به روش رقیق‌سازی، روش مؤثر و ساده‌ای می‌باشد. همچنین، راندمان این روش به حجم آب آلوده، حجم آب با نیترات پایین و نسبت اختلاط آن‌ها با یکدیگر بستگی دارد.

Title: ارزیابی روش‌های غربالگری سرطان پستان در زنان استان اصفهان

http://hsr.mui.ac.ir/index.php/jhsr/article/view/2732

مقدمه: سرطان پستان شایع‌ترین سرطان در زنان در سطح جهان و شایع‌ترین بدخیمی در بین زنان ایرانی می‌باشد که در سال‌های اخیر سیر صعودی قابل توجهی داشته است. روش‌ها: در مطالعه مقطعی حاضر، تعداد 9591 نفر از زنان 20 تا 65 سال شهرستان‌هاي تابعه دانشگاه علوم پزشكي اصفهان بررسی شد و داده‌ها با استفاده از پرسش‌نامه به صورت مصاحبه حضوری با مراجعه درب خانوار جمع‌آوری گردید. یافته‌ها: میانگین سنی زنان 7/11 ± 6/37 بود و 3/94 درصد از زنان متأهل و 9/91 درصد خانه‌دار بودند. 4/63 درصد خودآزمایی انجام می‌دادند و 5/61 درصد نداشتن مشکل و درد در ناحیه پستان را علت عدم انجام خودآزمایی ذکر نمودند. 2/50 درصد افراد، حداقل یک بار معاینه بالینی پستان داشتند و میانگین دفعات معاینه در سه سال گذشته 2/2 ± 9/1 بار به دست آمد. بیشترین علت عدم مراجعه جهت معاینه (9/85 درصد) نداشتن مشکل در پستان و کمترین مورد (2/4 درصد) هزینه‌بر بودن روش بود. 7/15 درصد افراد، ماموگرافی را حداقل یک بار تاکنون انجام داده‌اند و میانگین دفعات ماموگرافی 4/2 ± 3/2 بار بود 8/80 درصد افراد، علت عدم انجام ماموگرافی را نداشتن مشکل ذکر نمودند. بین انجام روش‌های غربالگری سرطان پستان با متغیرهای سن، شغل، سطح تحصیلات، تعداد فرزند، سابقه شیردهی و سابقه ابتلای خانوادگی ارتباط معنی‌دار آماری وجود داشت (001/0 > P برای همه). نتیجه‌گیری: وضعیت انجام روش‌های غربالگری در مطالعه جاری نسبت به مطالعات دیگر مطلوب است، ولی همچنان نیازمند ارتقا می‌باشد.

Title: بررسی انگ در مراقبان خانوادگی بیماران مبتلا به اختلالات روان‌پزشکی در بیمارستان فرشچیان همدان

http://hsr.mui.ac.ir/index.php/jhsr/article/view/3044

مقدمه: بی‌تردید رایج‌ترین و عمده‌ترین مسأله برای افراد مراقبت کننده از بیماران مبتلا به اختلالات روان‌پزشکی، انگ می‌باشد. هدف از انجام مطالعه حاضر، بررسی انگ در مراقبان خانوادگی بیماران دارای اختلالات روانی در بیمارستان فرشچیان همدان بود. روش‌ها: در این پژوهش مقطعی، 43 عضو خانواده مراقبت کننده از بیمار مبتلا به اختلال روانی در بیمارستان روان‌پزشکی فرشچیان شهر همدان، در سال 1394 به روش نمونه‌گیری در دسترس انتخاب شدند. ابزار سنجش، پرسش‌نامه 30 سؤالی محقق ساخته در مقیاس پنج درجه‌ای لیکرت بود که روایی آن بر اساس روش اعتبار محتوا و پایایی آن نیز از طریق محاسبه همبستگی درونی تأیید گردید. مقدار همبستگي دروني، 94/0 به دست آمد و نشان دهنده پايايي پرسش‌نامه در سطح بالا بود. داده‌ها با استفاده از آمار توصیفی در نرم‌افزار SPSS مورد تجزیه و تحلیل گرفت. یافته‌ها: از کل تعداد مراقبت کنندگان بیماران، 26 نفر (5/60 درصد) را زنان و 17 نفر (5/39 درصد) را مردان تشکیل دادند. میانگین نمره انگ، 23/12 ± 47/82 به دست آمد که نشان داد مراقبان خانوادگی رنج ناشی از انگی زندگی با بیمار روانی را تجربه کرده‌اند. بیشترین انگ مربوط به مواردی مانند عدم ازدواج، عدم یافتن کار، احساس شرمندگی، تحقیر شدن توسط دیگران، بی‌آبرویی و خجالت با نمرات بالا بود. نتیجه‌گیری: عدم آگاهی عمومی از اختلالات روانی و شناخت بیماران و کمبود مداخلات مؤثر، منجر به انگ می‌شود. با آگاهی از اختلالات روانی و علت‌یابی آن‌ها، می‌توان نقش این عوامل را در ایجاد انگ تعیین کرد. بدیهی است با درک و تدوین فرایندهای بهینه و اثربخش مانند آگاه کردن جامعه و آموزش، می‌توان به کاهش انگی عضو مراقبت کننده از بیماران دارای اختلال روانی کمک نمود.

Title: بررسی مصرف مکمل ویتامین D بر کاهش وزن، مقاومت انسولینی و پروتئین واکنشگر C در زنان چاق و دارای اضافه وزن شهر اصفهان

http://hsr.mui.ac.ir/index.php/jhsr/article/view/2924

مقدمه: کمبود ویتامین D به عنوان یک مشکل بهداشتی در سراسر جهان مطرح است. مطالعه حاضر با هدف بررسی تأثیر ویتامین D بر کاهش وزن، پروتئین واکنشگر C با حساسیت بالا (High sensitivity C-reactive protein یا hs-CRP) و مقاومت انسولینی زنان چاق و دارای اضافه وزن انجام شد. روش‌ها: این پژوهش از نوع کارآزمایی بالینی دو سوکور بود که بر روی 53 نفر از زنان چاق و دارای اضافه وزن صورت گرفت. نمونه‌ها به دو گروه تقسیم شدند. گروه مداخله، مکمل ویتامین D را با دوز 50000 واحد در هفته (به مدت 6 هفته) و گروه دیگر دارونما دریافت کرد. شاخص‌های آنتروپومتری و نشانگرهای بیوشیمیایی hs-CRP قبل و بعد از مداخله اندازه‌گیری گردید و با استفاده از آزمون‌های Paired t و Independent t مورد تجزیه و تحلیل قرار گرفت. یافته‌ها: پس از استفاده از مکمل ویتامین D، سطح سرمی ویتامین D افزایش معنی‌داری در مقایسه با گروه شاهد داشت (001/0 > P)، اما تفاوت معنی‌داری بین دو گروه در شاخص‌های آنتروپومتریک، سطح hs-CRP و مقاومت انسولینی مشاهده نشد (050/0 < P برای همه). نتیجه‌گیری: مکمل‌یاری ویتامین D با دوز 50000 واحد در هفته به مدت 6 هفته در زنان چاق و دارای اضافه وزن، تأثیری بر وزن، شاخص توده بدنی (Body mass index یا BMI)، دور کمر، مقاومت انسولینی و hs-CRP ندارد.

Title: اثر متقابل الگوهای غذایی و شاخص‌های تن‌سنجی با ابتلا به پوکی استخوان و کاهش تراکم استخوان

http://hsr.mui.ac.ir/index.php/jhsr/article/view/2957

مقدمه: پوکی استخوان (Osteoporosis)، از بزرگ‌ترین نگرانی‌های سلامت در دنیا محسوب می‌شود. با وجود نتایج مطالعات بسیاری مبنی بر تأثیر تغذیه بر سلامت استخوان، تاکنون تأثیر متقابل آن با عواملی مانند ژنتیک و شاخص‌های محیطی و تغذیه‌ای بررسی نشده است. هدف از انجام پژوهش حاضر، بررسی برهم‌کنش الگوهای غذایی و شاخص‌های تن‌سنجی [شاخص توده بدنی (Body mass index یا BMI)، نسبت دور کمر به دور باسن (Waist-hip ratio یا WHR)] با ابتلا به پوکی استخوان و کاهش تراکم استخوان بود. روش‌ها: دریافت‌های غذایی شرکت کنندگان (380 نفر) با استفاده از پرسش‌نامه تکرر مصرف مواد غذایی مورد سنجش قرار گرفت. تراکم مواد معدنی استخوانی (Bone mineral density یا BMD) نیز با استفاده از دستگاه تراکم‌سنجی در نواحی مهره‌های کمری و گردن ران ارزیابی گردید. شاخص‌های تن‌سنجی نیز با اندازه‌گیری قد، وزن، دور کمر و دور باسن محاسبه شد. یافته‌ها: پس از تعدیل مخدوشگر‌ها، برهم‌کنش میانه بالای الگوی غذایی سالم و چاقی عمومی (BMI بیشتر از 25 کیلوگرم بر مترمربع) در مدل سوم (مدل تعدیل شده برای سن، جنسیت، کالری، مصرف مکمل، استفاده از ضد آفتاب، فعالیت فیزیکی، تحصیلات و مصرف دخانیات) معنی‌دار و 43/0 (95/0-20/0) به دست آمد و در این افراد خطر ابتلا به پوکی استخوان و کاهش تراکم استخوان 57 درصد کاهش را نشان داد. خطر ابتلا به پوکی استخوان و کاهش تراکم استخوان در افرادی که در میانه پایین الگوی غذایی سنتی قرار داشتند و BMI بیشتر از 25 کیلوگرم بر مترمربع را دارا بودند، کمتر مشاهده شد. همچنین، خطر ابتلا در افرادی که چاقی عمومی داشتند و در میانه بالاتر از الگوی غذایی غربی قرار گرفتند، بیشتر بود. نتیجه‌گیری: اثر متقابل الگوی غذایی و شاخص‌های تن‌سنجی با ابتلا به پوکی استخوان و کاهش تراکم استخوان تنها در افرادی که چاقی عمومی داشتند و در میانه بالای مصرف الگوی غذایی سالم بودند، با کاهش خطر ابتلا همراه می‌باشد.

Title: افت جایگذاری ایرپلاگ در طول‌های مختلف کانال گوش شبیه‌سازی شده

http://hsr.mui.ac.ir/index.php/jhsr/article/view/3178

مقدمه: ایرپلاگ به عنوان یکی از تجهیزات حفاظتی سیستم شنوایی، عملکرد متفاوتی در فرکانس‌های مختلف دارد. پژوهش حاضر با شبیه‌سازی مدلی از کانال گوش انسان، تأثیر ایرپلاگ را در فرکانس و طول‌های مختلف کانال گوش مورد بررسی قرار داد. روش‌ها: در این مطالعه توصیفی- تحلیلی، در یک سمت کانال گوش شبیه‌سازی شده، میکروفون به عنوان دریافت‌ کننده صدا و در انتهای دیگر، ایرپلاگ تعبیه شد. پس از پخش صدای صورتی توسط بلندگو، میزان افت جایگذاری (Insertion loss یا IL) ایرپلاگ در فرکانس یک اکتاو و فواصل مختلف قرارگیری ایرپلاگ در کانال گوش شبیه‌سازی شده (8/12، 5/17، 5/25 و 1/31 میلی‌متر) بررسی و نتایج در نرم‌افزار LabVIEW ثبت گردید. یافته‌ها: یافته‌های میزان IL ایرپلاگ در فرکانس‌های یک اکتاو نشان داد که IL گوشی در فاصله 5/25 میلی‌متر در مقایسه با دیگر فواصل و در فرکانس‌های پایین (کمتر از 500 هرتز)، مقادیر بالاتری داشت و در این فاصله، در فرکانس حدود 4000 هرتز به بیشترین میزان رسید. نتیجه‌گیری: بالاترین میزان IL برای فرکانس‌های پایین در فاصله 5/25 میلی‌متر حاصل شد. متوسط طول کانال گوش انسان هم 5/2 سانتی‌متر است. بنابراین، بالاترین مقادیر تضعیف صدای محیط در سیستم شنوایی انسان نیز در همین طول صورت می‌گیرد. با توجه به حداکثر بودن میزان IL گوشی در این فاصله و در فرکانس 4000 هرتز، ساخت گوشی حفاظتی از مواد جاذب صدای این فرکانس پیشنهاد می‌شود.

Title: تأثیر سرما، ارتعاش و دستکش ضد ارتعاش بر میزان نیروی چنگش افراد در شرایط کنترل شده آزمایشگاهی

http://hsr.mui.ac.ir/index.php/jhsr/article/view/3062

مقدمه: اندازه‌گیری نیروی چنگش، یکی از شاخص‌های مهم علم ارگونومی به شمار می‌رود. عوامل مؤثر در میزان نیروی چنگش، گاهی باعث بروز Cumulative trauma disorder (CTD) در افراد می‌شود. مطالعه حاضر با هدف بررسی میزان تأثیر دستکش‌های ضد ارتعاش، سرما و ارتعاش بر روی نیروی چنگش انجام گرفت. روش‌ها: این پژوهش از نوع مقطعی بود که بر روی 30 نفر از کارکنان دانشگاه علوم پزشکی اصفهان انجام شد. به منظور اندازه‌گیری نیروی چنگش، از داینامومتر مدل JAMAR مطابق توصیه انجمن درمانگران دست آمریکا (American Society of Hand Therapists یا ASHT) استفاده گردید. برای دستیابی به میزان نیروی چنگش، از افراد درخواست شد که با تمام قدرت دسته داینامومتر را فشار دهند. سپس میزان نیروی چنگش بر حسب کیلوگرم نیرو از روی صفحه نمایش یادداشت گردید. به منظور بررسی اثر توأم عوامل مذکور بر روی قدرت چنگش، 18 حالت ترکیبی مورد سنجش قرار گرفت. یافته‌ها: رابطه معکوسی بین میزان ارتعاش و قدرت چنگش مشاهده شد. همچنین، میزان قدرت چنگش افراد در دماهای پایین، کاهش یافت. دستکش‌های ضد ارتعاش مختلف نیز تأثیرات متفاوتی بر کاهش نیروی چنگش داشت. سه عامل سرما، ارتعاش و دستکش تأثیر معنی‌داری را بر قدرت چنگش نشان داد (050/0 > P) و ضریب همبستگی این عوامل به ترتیب 172/0، 363/0- و 602/0- به دست آمد. نتیجه‌گیری: پوشیدن دستکش بیشترین تأثیر را بر کاهش نیروی چنگش دارد. پس از آن، عامل ارتعاش نیز به عنوان دومین عامل مؤثر کاهش دهنده در میزان نیروی چنگش شناخته شد. همچنین مشخص گردید که با کاهش دما، میزان نیروی چنگش کاهش می‌یابد.

Title: بررسی عملکرد کامپوزیت نانولوله کربنی/ کیتوسان در جذب آفت‌کش دیازینون از پساب‌های کشاورزی

http://hsr.mui.ac.ir/index.php/jhsr/article/view/3060

مقدمه: امروزه آلودگي منابع آب به وسيله سموم آفت‌كش و وجود باقی‌مانده آن‌ در زنجیره غذایی، يكي از معضلات زيست محيطي محسوب می‌گردد. بنابراین، حذف آفت‌کش‌ها در طی فرایند تصفیه آب، اهمیت فراوانی دارد. پژوهش حاضر با هدف بررسی جذب آفت‌کش دیازینون توسط جاذب نانولوله کربنی/ کیتوسان (Chitosan/Carbon Nanotube یا CHN-CNT) انجام شد. روش‌ها: جاذب CHN-CNT از روش پیوند بین زنجیره پلیمری (Cross linking methods) با نسبت‌های وزنی مختلف نانولوله کربنی (Carbon Nanotube یا CNT) به کیتوسان به دست آمد. به منظور بررسی مورفولوژی و ساختار شیمیایی جاذب سنتز شده، از میکروسکوپ الکترونی روبشی (Scanning electron microscopy یا SEM) و طیف‌سنج مادون قرمز (Fourier-transform infrared spectroscopy یا FTIR) استفاده گردید. همچنین، تأثیر شاخص‌های میزان غلظت جاذب، غلظت دیازینون و زمان تماس بر میزان جذب دیازینون روی CHN-CNT مورد بررسی قرار گرفت. یافته‌ها: جاذب CHN-CNT تا حدود زیادی دیازینون را از محیط آبی حذف نمود؛ به طوری که ظرفیت جذب دیازینون (qe) در غلظت 50 میلی‌گرم بر لیتر دیازینون و غلظت 5/0 گرم بر لیتر جاذب و زمان 65 دقیقه، به 59 میلی‌گرم به گرم جاذب رسید. فرایند احیای جاذب با چهار چرخه متوالی انجام گرفت. درصد احیای جاذب در چرخه چهارم، 9/94 درصد به دست آمد. نتیجه‌گیری: استفاده از جاذب CHN-CNT به دلیل ساخت آسان، کارایی بالا در جذب و قابلیت احیای مؤثر وآسان، می‌تواند به عنوان جاذب مناسبی در حذف آفت‌کش‌ها از منابع آبی آلوده مورد استفاده قرار گیرد.

Title: بررسی کارایی خاک رس اصلاح شده با سورفکتانت کاتیونی در حذف نیترات از محلول‌های آبی

http://hsr.mui.ac.ir/index.php/jhsr/article/view/3073

مقدمه: امروزه افزایش غلظت نیترات در آب‌های سطحی و زیرزمینی به علت افزایش تولید فاضلاب‌ها و گسترش فعالیت‌های کشاورزی، یکی از مشکلات محیطی در بسیاری از مناطق جهان از جمله ایران به شمار می‌رود. مصرف آب حاوی غلظت بالای نیترات، مشکلاتی را در زمینه محیط زیست و بهداشت عمومی ایجاد می‌کند. هدف از انجام مطالعه حاضر، بررسی کارایی مونتموریلونیت اصلاح شده با سورفکتانت کاتیونی هگزادسیل تری‌متیل آمونیوم بروماید در حذف نیترات از محلول‌های آبی بود. روش‌ها: این تحقیق از نوع تجربی- آزمایشگاهی بود و به منظور حذف نیترات از محلول‌های آبی از طریق خاک رس اصلاح شده با سورفکتانت کاتیونی در یک سیستم بسته انجام شد. تأثیر شاخص‌های مختلف مانند غلظت اولیه نیترات، دوز جاذب، pH و زمان تماس بر راندمان حذف نیترات با استفاده از نرم‌افزار طراحی آزمایش (Design of experiments یا DOE) مورد بررسی قرار گرفت. یافته‌ها: شرایط بهینه حذف نیترات در غلظت اولیه نیترات 200 میلی‌گرم بر لیتر، دوز جاذب 5/7 گرم بر لیتر، pH برابر با 7 و زمان تماس 40 دقیقه به دست آمد. در این شرایط، راندمان حذف نیترات 27/71 درصد و ظرفیت جذب نیترات 06/19 میلی‌گرم بر گرم بود. نتیجه‌گیری: می‌توان از خاک رس اصلاح شده با سورفکتانت کاتیونی، به عنوان جاذب مؤثری در جهت حذف نیترات از محلول‌های آبی استفاده نمود.

Title: اثر غلظت اسانس‌های روغنی آویشن و رزماری و طول زمان نگهداری بر خواص کیفی قارچ تکمه‌ای

http://hsr.mui.ac.ir/index.php/jhsr/article/view/3115

مقدمه: قارچ تکمه‌ای به دلیل عدم وجود کوتیکول، سرعت بالای تنفس، رطوبت زیاد و فعالیت شدید آنزیمی، به سرعت و بلافاصله پس از برداشت، فاسد و تغییر رنگ آن آغاز می‌شود. هدف از انجام مطالعه حاضر، بررسی تأثیر اسانس‌های روغنی گیاهان دارویی آویشن و رزماری بر برخی خصوصیات کیفی قارچ تکمه‌ای بود. روش‌ها: این پژوهش در قالب یک طرح تصادفی انجام شد و تأثیر غلظت‌های صفر، 5/2، 5، 5/7 و 10 میکرولیتر بر لیتر اسانس‌های رزماری و آویشن و زمان‌های صفر، 5، 10، 15 و 20 روز را بر خصوصیات کیفی قارچ تکمه‌ای شامل سفتی بافت، رطوبت اندام باردهی، شاخص قهوه‌ای شدن و ظرفیت آنتی‌اکسیدانی بررسی نمود. یافته‌ها: با افزایش مدت زمان نگهداری، رطوبت و سفتی بافت نسبت به تیمار شاهد کاهش یافت. بیشترین رطوبت در غلظت‌های صفر و 5 میکرولیتر بر لیتر اسانس‌ها به میزان 5/13 و 0/13 درصد و بیشترین سفتی به ترتیب در غلظت‌های 5/7، 5، 10 و 5/2 میکرولیتر بر لیتر اسانس آویشن با میزان 95/10، 52/10، 90/9 و 87/9 درصد به دست آمد. بیشترین آنتی‌اکسیدان نیز در غلظت‌های مورد بررسی در روزهای صفر، 5 و 10 در بازه‌ای بین 60 تا 75 درصد مشاهده شد. همچنین، با افزایش مدت زمان نگهداری نسبت به روز صفر، شاخص قهوه‌ای شدن افزایش معنی‌داری را نشان داد. نتیجه‌گیری: کاربرد اسانس‌ها با توجه به نوع، غلظت و مدت زمان نگهداری محصول، نتایج متفاوتی را بر خصوصیات کیفی قارچ تکمه‌ای نشان داد که با توجه به هدف مورد بررسی می‌توان غلظت مناسب را انتخاب نمود. بر اساس نتایج به دست آمده، به نظر می‌رسد که غلظت 5 میکرولیتر بر لیتر اسانس آویشن، تیمار مناسبی جهت حفظ خصوصیات کیفی اندام باردهی قارچ تکمه‌ای در طول دوره نگهداری می‌باشد.

Title: ارزیابی مقدار فلزات سنگين ارسنیک، كادميوم و سرب در سيب زميني در اراضي جنوب شرقي اصفهان در سال 1395

http://hsr.mui.ac.ir/index.php/jhsr/article/view/3096

مقدمه: هدف از انجام مطالعه حاضر، ارزیابی میانگین غلظت فلزات سنگین (سرب، کادمیوم و آرسنیک) در سیب‌زمینی مزارع جنوب شرقی اصفهان بود. روش‌ها: نمونه‌های خاک، سیب‌زمینی، کود و آب به روش هضم اسیدی با استفاده از کتاب روش‌های استاندارد به همراه نمونه استاندارد هضم گردید. سپس میزان فلزات سنگین سرب، کادمیوم و آرسنیک با استفاده از دستگاه جذب اتمي مجهز به كوره گرافيتي تعیین شد. جهت تعیین ارتباط غلظت‌های متفاوت فلزات سنگين، آزمون ANOVA در نرم‌افزار SPSS مورد استفاده قرار گرفت. یافته‌ها: میانگین غلظت فلزات سرب، آرسنیک و کادمیوم در آب آبیاری پایین‌تر از حد استاندارد مشخص گردید. میانگین غلظت آرسنیک، سرب و کادمیوم در نمونه‌های خاک به ترتیب 6072/2، 7417/1 و 5693/0 میلی‌گرم بر کیلوگرم به دست آمد که کمتر از حد استاندارد بود. میانگین غلظت فلزات سنگین در کود مصرفی نیز پایین‌تر از حد استاندارد بود. بالاترین میانگین غلظت فلز سنگین در سیب‌زمینی، به فلز سرب با میزان 8074/4 میلی‌گرم بر کیلوگرم اختصاص داشت که بالاتر از حد استاندارد می‌باشد و غلظت فلزات کادمیوم و آرسنیک به ترتیب 6923/2 و 7869/1 میلی‌گرم بر کیلوگرم گزارش شد که کادمیوم بالاتر از حد استاندارد است. فاکتور انتقال (Transfer factor) از الگوی آرسنیک < سرب < کادمیوم پیروی نمود و مقادیر آن به ترتیب 7291/4 (کادمیوم)، 7601/2 (سرب) و 6853/0 (آرسنیک) به دست آمد.نتیجه‌گیری: غلظت کادمیوم و سرب در سیب‌زمینی بالاتر از حد استاندارد می‌باشد که می‌توان آن را به تحرک بالای این دو فلز در خاک و انتقال آن به گیاه نسبت داد. در نتیجه، مصرف سیب‌زمینی تولیدی در منطقه مورد بررسی ممکن است در آینده خطر سلامتی را در جمعیت محلی مصرف‌کننده افزایش دهد.

Title: بررسی عوامل مؤثر بر مصرف روغن زیتون: مطالعه نواحی شهری و روستایی کشور

http://hsr.mui.ac.ir/index.php/jhsr/article/view/3116

مقدمه: اهمیت مصرف روغن زیتون از بعد غذایی و اقتصادی، مطالعه در خصوص عوامل مؤثر بر مصرف آن در بین مناطق شهری و روستایی کشور را با هدف برنامه‌ریزی و ارزیابی سیاست‌های غذایی و سلامت کشور ضروری ساخت. روش‌ها: مطالعه حاضر، با بهره‌گیری از اطلاعات اقتصادی و جمعیتی خانوارهای شهری و روستایی کشور طی سال‌های 1394-1383 که از گزارش‌های مرکز آمار ایران استخراج شد، نقش عوامل قیمتی و غیر قیمتی مؤثر در مصرف روغن زیتون بر اساس رویکرد داده‌های ادغام شده، مورد بررسی قرار گرفت. یافته‌ها: آزمون F Limer، برتری مدل تلفیقی بر مدل ترکیبی را نشان داد. بر این اساس، مدل با این رویکرد و نیز با توجه به وجود ناهمسانی واریانس، به صورت حداقل مربعات تعمیم یافته (GLS یا Generalized least square) برآورد شد. بر اساس مقدار ضرایب برآوردی در هر دو الگوی مربوط به خانوارهای شهری و روستایی، عوامل غیر قیمتی همچون سن، سطح تحصیلات و اشتغال سهم بیشتری از مصرف در مقایسه با قیمت و بودجه داشت. نتیجه‌گیری: توجه به سیاست‌هایی چون آگاهی افراد جامعه در مورد مصرف روغن زیتون و تمرکز آموزش روی همه افراد جامعه با سطوح تحصیلات مختلف مانند آموزش همگانی در مدارس، دانشگاه‌ها و خانه‌های بهداشت، در کنار بهبود سیاست‌گذاری‌ها در جهت سازمان‌دهی مناسب و رفع نواقص موجود در توزیع و فروش این محصولات، به گونه‌ای که امکان دسترسی بیشتر اقشار مختلف جامعه به این نوع روغن فراهم گردد، اهمیت داشته و پیشنهاد می‌شود.

Title: تبیین مؤلفه‌های روان‌شناختی سبک زندگی سلامت‌ محور: یک مطالعه کیفی

http://hsr.mui.ac.ir/index.php/jhsr/article/view/3029

مقدمه: سبک ‌زندگی سلامت‌محور یکی از موضوعات مهم حوزه سلامت است و شناسایی ابعاد گوناگون آن از اهمیت ویژه‌ای برخوردار است. بر این اساس، پژوهش حاضر با هدف تبیین مؤلفه‌های روان‌شناختی سبک زندگی سلامت‌محور انجام شد. روش‌ها: مطالعه حاضر با رویکرد پژوهش کیفی و با روش تحلیل محتوای کیفی انجام شد. بدین منظور، 16 نفر که دارای سبک زندگی سالم بودند به روش نمونه‌گیری هدفمند انتخاب شده و با استفاده از مصاحبه عمیق مورد مطالعه قرار گرفتند. تحلیل داده‌ها بر اساس مراحل تحلیل محتوای Graneheim و Landman صورت گرفت. یافته‌ها: 4 مقوله و 15 زیرمقوله شناسایی شد که عبارت از ویژگی‌های شخصیتی (اراده و پشتکار، هدفمندی، انعطاف‌پذیری و قاطعیت)، باورها (سودمندی روابط اجتماعی، مدیریت‌پذیری زندگی و ارزشمندی سلامت)، توانمندی‌ها (مسؤولیت‌پذیری، شوخ‌طبعی، خوش‌بینی، خودکارامدی و خودآگاهی) و نگرش‌ها (نگرش نسبت به خود، نگرش نسبت به رفتارهای سلامت و نگرش نسبت به رفتارهای آسیب‌رسان سلامت) بود. نتیجه‌گیری: در پژوهش حاضر مؤلفه‌های روان‌شناختی سبک زندگی سلامت‌محور مورد بررسی قرار گرفت و نتایج نشان داد که ویژگی‌های شخصیتی، باورها، توانمندی‌ها و نگرش‌ها در سبک زندگی سلامت‌محور نقش دارد. بنابراین، پیشنهاد می‌شود که در پژوهش‌ها و آموزش‌های آینده بیشتر مورد توجه قرار گیرد.

Title: معرفی روش میکرواستخراج تک قطره در فضای فوقانی و آنالیز گاز کروماتوگرافی برای ارزیابی نمونه‌های سنتتیک فرمالدئید

http://hsr.mui.ac.ir/index.php/jhsr/article/view/2776

مقدمه: با توجه به استفاده گسترده از فرمالدئید در صنایع و مراکز پزشکی، سمیت آن و نگرانی جهت حساسیت و دقت روش آنالیز این ترکیب، مطالعه حاضر با هدف بررسی هم‌زمان استفاده از حساس‌ترین روش توصیه شده تعیین فرمالدئید توسط مؤسسه ملی بهداشت و ایمنی شغلی (National Institute of Occupational Safety and Health یا NIOSH) و کاربرد آزمایشگاهی روش میکرواستخراج تک قطره در فضای فوقانی (Headspace-Single drop Microextraction یا HS-SDME) در آماده‌سازی نمونه‌های حاوی فرمالدئید با آنالیز گاز کروماتوگرافی (Gas chromatography یا GC) در نمونه‌های سنتتیک انجام شد. روش‌ها: در معرفی روش HS-SDME و آنالیز GC جهت اندازه‌گیری فرمالدئید، نمونه‌های مورد نياز با غلظت‌های معین با استفاده از دو روش NIOSH 3500 و HS-SDME آماده‌سازی و تجزیه و تحلیل تحلیل شدند و نتایج دو روش مورد مقایسه قرار گرفت. یافته‌ها: بررسی صحت HS-SDME، با مقایسه نتایج دو روش انجام شد. میزان تکرارپذیری و بازده استخراج روش HS-SDME با روش Occupational Safety and Health Administration (OSHA) قابل مقایسه بود. همچنین، روش HS-SDME دارای صحت بود. نتیجه‌گیری: روش HS-SDME در کاربرد آزمایشگاهی دقت و صحت لازم را دارد. همچنین، بازده استخراج روش HS-SDME با روش OSHA جهت اندازه‌گیری فرمالدئید قابل مقایسه می‌باشد. روش HS-SDME به مطالعه و بررسی بیشتری نیازمند است.

Title: کاربرد تاریخ پژوهی محلی در تبیین پراکندگی شیوع بیماری Multiple Sclerosis در ایران (مطالعه موردی: منطقه پیربکران، تابستان 1395)

http://hsr.mui.ac.ir/index.php/jhsr/article/view/3061

مقدمه: از اهداف مهم تاریخ پژوهی محلی پرداختن به موضوعاتی است که با زندگی روزمره انسان‌ها در ارتباط باشد. پژوهش حاضر به تبيين پراکندگی، شيوع بيماري Multiple Sclerosis (MS) در ایران با تمرکز بر منطقه پيربکران اصفهان پرداخت که یکی از کانون‌های MS می‌باشد. روش‌ها: در پژوهش حاضر سعی شد که علاوه بر استفاده از تحقیقات کتابخانه‌ای به روش میدانی و با تکنیک تاریخ شفاهی با نمونه آماری، با 25 نفر از بیماران مبتلا به MS مصاحبه شود و شرح حال آن‌ها قبل از مبتلا شدن به بیماری مورد بررسی قرار گیرد تا مشخص شود کدام یک از عوامل احتمالی باعث بروز و ظهور MS در افراد مختلف شده است. سؤال اصلی این بود که آیا شیوع MS در تمام نقاط ایران پراکندگی یکسان دارد؟ با این فرضیه که MS در برخی نواحی و در نژادهایی که مستعد هستند، شیوع بیشتری دارد. یافته‌ها: نتایج تحقیقات میدانی در منطقه پیربکران نشان داد که اولین بیمار MS مربوط به 45 سال پیش است که متقارن با فعالیت‌های انسانی در جهت تغییر محیط زیست از یک منطقه کشاورزی به یک منطقه صنعتی می‌باشد. بنابراین، با توجه به این که در بین افراد مهاجر فرد مبتلا به MS شناسایی نشد، می‌توان نتیجه‌گیری نمود که اهالی منطقه پیربکران از نظر ژنتیکی مستعد بیماری هستند. نتیجه‌گیری: عوامل مؤثر بر بروز بیماری MS چند وجهی می‌باشد. بر اساس نتایج پژوهش، افرادی که از نظر ژنتیکی مستعد ابتلا به MS می‌باشند، با مهاجرت به مناطق با خطر کم، می‌توانند از مبتلا شدن به این بیماری جلوگیری نمایند.

Title: بررسی عوامل پیشگویی‌کننده خودمراقبتی،آگاهی و باور عمومی در مصرف منطقی آنتی‌بیوتیک‌ها: کاربرد تئوری شناختی- اجتماعی

http://hsr.mui.ac.ir/index.php/jhsr/article/view/3241

مقدمه: افزایش مقاومت آنتی‌میکروبی، نتیجه افزایش مصرف آنتی‌بیوتیک در مواقع غیر ضروری است. گونه‌های مقاوم و کاهش کارایی آنتی‌بیوتیک‌ها باعث تحمیل هزینه‌های زیادی به سیستم سلامت شده است. پژوهش حاضر، با هدف تعیین عوامل پیش‌بینی کننده خودمراقبتی، آگاهی و باور عمومی در مصرف منطقی آنتی‌بیوتیک‌ها بر اساس تئوری شناختی- اجتماعی در مراجعان مراکز بهداشتی درمانی جنوب تهران طراحی و اجرا شد. روش‌ها: مطالعه حاضر تحلیلی با مشارکت 260 نفر از مراجعان به مراکز بهداشتی درمانی جنوب تهران در سال 1396 انجام شد که به روش تصادفی از 8 مرکز تابعه دانشگاه وارد مطالعه شدند. ابزار جمع‌آوری اطلاعات، پرسش‌نامه محقق ساخته بر طبق تئوری شناختی- اجتماعی بود که شامل اطلاعات جمعیتی شرکت کنندگان و پرسش‌نامه سؤالات خودمراقبتی، آگاهی و باورعمومی می‌شد و به روش خودگزارش‌دهی تکمیل گردید. برای تحلیل داده‌ها، از نرم‌افزار SPSS، آزمون‌های رگرسیون خطی و ضریب همبستگی Pearson استفاده شد. سطح معنی‌داری در مطالعه حاضر کمتر از 05/0 در نظر گرفته شد. یافته‌ها: میانگین و انحراف معیار سنی شرکت کنندگان 4/12 ± 3/36 سال بود. از میان سازه‌های تئوری شناختی- اجتماعی، دانش، خودکنترلی و خودمدیریتی سهم معنی‌داری در تبیین خودمراقبتی داشت (05/0 > P). سازه دانش تنها سازه‌ای بود که آگاهی را پیش‌بینی کرد (05/0 > P). سازه دانش، انتظارات پیامد، موانع درک شده و سازگاری عاطفی سازه‌هایی بود که باور عمومی را تبیین نمود (05/0 > P). نتیجه‌گیری: مطالعه حاضر نشان داد که سازه‌های تئوری شناختی- اجتماعی می‌تواند جهت طراحی مداخلات آموزشی برای بهبود خودمراقبتی، آگاهی و باور عمومی در زمینه مصرف منطقی آنتی‌بیوتیک‌ها مؤثر باشد.

Title: اثربخشی درمان مبتنی بر پذیرش و تعهد بر افسردگی و حرمت خود در زنان مبتلا به اختلال افسردگی عمده

http://hsr.mui.ac.ir/index.php/jhsr/article/view/3202

مقدمه: شیوع افسردگی در زنان به طور تقریبی دو برابر مردان می‌باشد؛ به طوری که پیش‌بینی شده تا سال 2020 افسردگی شایع‌ترین و پرهزینه‌ترین بیماری خواهد بود. بنابراین، هدف از انجام پژوهش حاضر، تعیین اثربخشی درمان مبتنی بر پذیرش و تعهد بر افسردگی و حرمت خود در زنان مبتلا به اختلال افسردگی عمده بود. روش‌ها: پژوهش حاضر به صورت آزمایشی با طرح تک‌ موردی انجام گرفت. جامعه شامل زنان مبتلا به اختلال افسردگی عمده و بستری در بیمارستان فارابی اصفهان بود. 5 نفر به صورت تصادفی ساده انتخاب شدند. پس از سه هفته دارو درمانی، بیماران به مدت ده جلسه فردی تحت درمان قرار گرفتند. ابزار جمع‌آوری داده‌ها پرسش‌نامه افسردگی Beck و حرمت خود Rozenberg بود. پیش از شروع مطالعه و در جلسات ششم و دهم، پرسش‌نامه‌ها تکمیل شد. بررسی در مرحله پیگیری هم پس از گذشت دو ماه انجام شد.جهت تجزیه و تحلیل داده‌ها، از نرم‌افزار SPSS، روش‌های تحلیل روند، ترسیم نمودار و شاخص بهبودی بالینی استفاده شد. یافته‌ها: نمره‌های افسردگی بیماران در مرحله پس‌آزمون (بهبودی 76/84 درصد) و پیگیری (بهبودی 24/88 درصد) کاهش و نمره‌های حرمت خود در مرحله پس‌آزمون (بهبودی 44/63 درصد) و پیگیری (بهبودی 18/69 درصد) افزایش یافت‌. نتیجه‌گیری: نتایج پژوهش حاضر تأثیر و کارایی درمان مبتنی بر پذیرش و تعهد را بر کاهش افسردگی و افزایش حرمت خود بیماران مبتلا به افسردگی عمده مورد تأیید قرار داد. همچنین، بیانگر این است که از این درمان برای بیماران مبتلا به افسردگی می‌توان استفاده نمود.
